# Investigation of the Mechanism Underlying Calcium Dobesilate-Mediated Improvement of Endothelial Dysfunction and Inflammation Caused by High Glucose

**DOI:** 10.1155/2019/9893682

**Published:** 2019-10-21

**Authors:** Yijun Zhou, Chaojun Qi, Shu Li, Xinghua Shao, Zhaohui Ni

**Affiliations:** Department of Nephrology, Ren Ji Hospital, School of Medicine, Shanghai Jiaotong University, Shanghai, China

## Abstract

**Background/Aims:**

Diabetic kidney disease (DKD) is a leading cause of end-stage renal disease. Calcium dobesilate (CaD) is widely used to treat diabetic retinopathy. Recent studies have demonstrated that CaD exerts protective effects against diabetic nephropathy. The aim of this study was to elucidate the molecular and cellular mechanisms underlying the protective effects of CaD.

**Methods:**

Human umbilical vein endothelial cells (HUVECs) were cultured with different D-glucose concentrations to determine the effects of high glucose on HUVEC gene expression. HUVECs were also incubated with CaD (25 *μ*M, 50 *μ*M, and 100 *μ*M) for 3 days to determine the effects of CaD on HUVEC viability. db/db mice were treated with CaD. 2-[(Aminocarbonyl)amino]-5-(4-fluorophenyl)-3-thiophenecarboxamide (TPCA-1) blocked the nuclear factor-*κ*B (NF-*κ*B) pathway in HUVECs. A pentraxin 3 (PTX3) small interfering RNA (siRNA) intervention experiment was performed in the cells. An adenovirus-encapsulated PTX3 siRNA intervention experiment was performed in db/db mice. Western blot and real-time PCR analyses were used to detect PTX3, p-IKBa/IKBa (I-kappa-B-alpha), and p-eNOS/eNOS (endothelial nitric oxide synthase) expression in mice and HUVECs. Hematoxylin-eosin (HE) staining and periodic acid-Schiff (PAS) staining were used to observe renal tissue damage in mice. PTX3 expression was observed by immunohistochemical staining.

**Results:**

CaD downregulated the expression of PTX3 and p-IKBa/IKBa and upregulated the expression of p-eNOS/eNOS in vitro. When TPCA-1 was used, high glucose induced high PTX3 expression, and the expression of p-eNOS/eNOS increased. After PTX3 gene silencing, the expression of p-eNOS/eNOS also increased. In vivo, CaD reduced the expression of PTX3 and p-IKBa/IKBa in the kidneys of db/db mice and increased the expression of p-eNOS/eNOS. After PTX3 gene silencing, the urine protein and renal function of db/db mice were ameliorated, the glomerular extracellular matrix was decreased, and the expression of p-eNOS/eNOS was increased.

**Conclusions:**

Our results suggested that CaD may inhibit the expression of PTX3 by altering the IKK/IKB/NF-*κ*B pathway, thereby improving endothelial dysfunction in HUVECs. PTX3 may be a potential therapeutic target for DKD.

## 1. Introduction

In developed countries, diabetic kidney disease (DKD) is the leading cause of chronic kidney disease [1]. The pathogenesis of DKD is unclear. Endothelial dysfunction and microinflammation are the two major pathogenic mechanisms of diabetic nephropathy [2–7].

Pentraxin 3 (PTX3) is an important component of humoral immunity. PTX3 is secreted by various cells, including endothelial cells, mononuclear macrophages, fibroblasts, adipocytes, dendritic cells, and smooth muscle cells, after inflammatory stimulation [8–10]. High plasma PTX3 levels are associated with many human diseases related to endothelial dysfunction, including chronic kidney disease, preeclampsia, and hypertension-related multisystem complications [11–14]. In a model of superior mesenteric ischemia-reperfusion injury in PTX3^−/−^ mice, tissue damage was alleviated, while nuclear factor-*κ*B (NF-*κ*B) transport, cytokine CXCL1 production, and tumor necrosis factor accumulation were all reduced [15]. Studies have shown that palmitic acid promotes PTX3 expression through the IKK/IKB/NF-*κ*B pathway, thereby enhancing apoptosis and initiating inflammatory responses [16]. PTX3 interferes with the phosphorylation of endothelial nitric oxide synthase (eNOS) at Serine 1177 to reduce nitric oxide production, leading to endothelial cell dysfunction [17]. Therefore, PTX3 may be a bridge between inflammation and endothelial dysfunction.

Calcium dobesilate (CaD) is widely used to treat diabetic retinopathy and improves systemic hemodynamics [18]. Moreover, studies have suggested that CaD inhibits high glucose-induced NF-*κ*B pathway activation in diabetic retinopathy [19]. Studies have found that CaD also has therapeutic effects on microalbuminuria in type 2 diabetes [20]. However, the mechanism of action of CaD in diabetic nephropathy is still unclear.

The present study is aimed at investigating whether high glucose promotes the expression of PTX3 through the IKK/IKB/NF-*κ*B pathway, thereby interfering with the phosphorylation of eNOS, leading to endothelial cell dysfunction. The present study is also aimed at testing whether CaD exerts its protection through the NF-*κ*B pathway.

## 2. Subjects and Methods

### 2.1. Human Umbilical Vein Endothelial Cell (HUVEC) Culture

Primary HUVECs and endothelial cell growth medium (ECM) were purchased from ScienCell Research Laboratories (6076 Corte Del Cedro, Carlsbad, CA 92011). Culture flasks were coated with poly-l-lysine before use, and the cells were cultured in ECM supplemented with ECM growth supplement, 5% fetal bovine serum, and penicillin/streptomycin solution at 37°C in 5% CO_2_. HUVECS at the 2nd to 5th passages were used in all experiments. We followed the methods of Zhou et al. [21].

### 2.2. Real-Time Polymerase Chain Reaction (RT-PCR)

Total RNA was extracted after medium removal using TRIzol reagent (TaKaRa Bio, Inc., Otsu, Japan) according to the manufacturer's instructions. PTX3 mRNA expression was analyzed via RT-PCR using SYBR Green Master Mix (TaKaRa Bio, Inc., Otsu, Japan). One microgram of RNA was reverse-transcribed to cDNA using random primers (Life Technologies BRL, Grand Island, NY, USA) and Moloney murine leukemia virus (MMLV) reverse transcriptase (TaKaRa Bio, Inc., Otsu, Japan). Real-time PCR was performed using an Applied Biosystems TaqMan 7000 system (Applera, Darmstadt, Germany) in 384-well plates containing 5 *μ*L reaction mixtures. GAPDH was tested as the internal control, and the data are expressed as fold increases in mRNA expression. All primers are listed in Table 1.

### 2.3. Western Blot Analysis

SDS-PAGE and immunoblot analyses were carried out according to standard protocols and visualized using chemiluminescent HRP substrate (Millipore Corporation, Billerica, MA, USA). Nitrocellulose filter membranes were incubated at 4°C overnight with the following primary antibodies: anti-PTX3 (1 : 600 Proteintech 13797-1-AP), anti-IKB*α* (1 : 10000 Abcam ab32518), anti-IKB*α* (phospho S36) (1 : 1000 Abcam ab133462), anti-eNOS (phospho S1177) (1 : 500 Abcam ab195944), and anti-eNOS (1 : 1000 Abcam ab199956).

### 2.4. RNA Interference

Three siRNA targets were designed according to the transcript of mouse PTX3 gene, and primer synthesis was arranged. The single-stranded primers were annealed into double-stranded oligo sequences, which were linked to RNA interference vectors linearized by double digestion and replaced the original ccdB toxicity gene. The transformants were screened by colony PCR, and the positive clones were sequenced. The correct clone was verified by sequencing, and high purity plasmid was extracted. Small interfering RNAs (siRNAs) were synthesized by GenePharma (Shanghai, China) and transfected into HUVECs using Lipofectamine 2000 to suppress the function of PTX3 (Invitrogen).

### 2.5. Animal Experimental Subjects and Grouping

The db/db mice (male, SPF grade, 8 weeks old) and db/m mice (male, SPF grade, 8 weeks old) were purchased from Shanghai SLAC Laboratory Co., Ltd. All experimental mice were housed in SPF-class animal rooms, fed freely, and provided a 12-hour light-dark environment.

Eight-week-old db/db mice were administered intragastric CaD (100 mg/kg) and the same amount of saline. db/m mice were used as the control. Every group contained eight mice. The mice were killed at 12 and 16 weeks of age. In the adenovirus intervention experiment, adenovirus (2 × 108 PFU) and the same amount of saline were injected into the tail veins of the 8-week-old db/db mice every two weeks, while db/m mice were used as the control. Every group contained eight mice. The mice were sacrificed at 16 weeks of age. The construction of the mouse siRNA plasmid and adenovirus packaging was designed by Heyuan Biotechnology (Shanghai) Co., Ltd.

### 2.6. Statistical Analysis

Continuous variables are expressed as the mean values (mean ± standard error of the mean). A *t*-test was used to compare the two groups. One-way analysis of variance was used for the comparative analysis in multiple groups followed by LSD post hoc tests. *P* < 0.05 was considered to indicate statistically significant differences. All data were analyzed by SPSS 20.0 statistical software (version 20.0; IBM SPSS, Armonk, NY, USA).

## 3. Results

### 3.1. In Vitro Studies

#### 3.1.1. High Glucose Increases PTX3 and p-IKBa/IKBa Expression but Decreases p-eNOS/eNOS Expression in HUVECs

**Figure 1:**
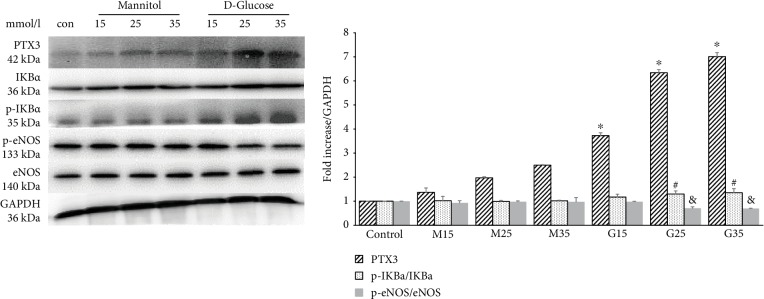
The expression of PTX3, p-IKBa/IKBa, and p-eNOS/eNOS with different concentrations of glucose in HUVEC; con: control; M15: mannitol 15 mmol/L; M25: mannitol 25 mmol/L; M35: mannitol 35 mmol/L; G15: D-glucose 15 mmol/L; G25: D-glucose 25 mmol/L; G35: D-glucose 35 mmol/L; ^∗^^, &, #^compared with the control group, *P* < 0.05.

HUVECs were cultured for 48 hours with different concentrations of D-glucose (15, 25, and 35 mmol/L), and the control group was cultured with a glucose concentration of 5.5 mmol/L. Additionally, mannitol was used as an osmotic pressure control, and the mannitol concentrations were 15, 25, and 35 mmol/L. As shown in Figure 1, the protein expression of PTX3 and p-IKBa/IKBa gradually increased as the concentration of glucose increased in a concentration-dependent manner. However, there was no significant change in the mannitol groups. In addition, high glucose decreased the p-eNOS/eNOS levels in endothelial cells (Figure 1).

#### 3.1.2. CaD Inhibits the Increased Expression of PTX3 and p-IKBa/IKBa but Enhances the Decreased Expression of p-eNOS/eNOS Induced by High Glucose

**Figure 2:**
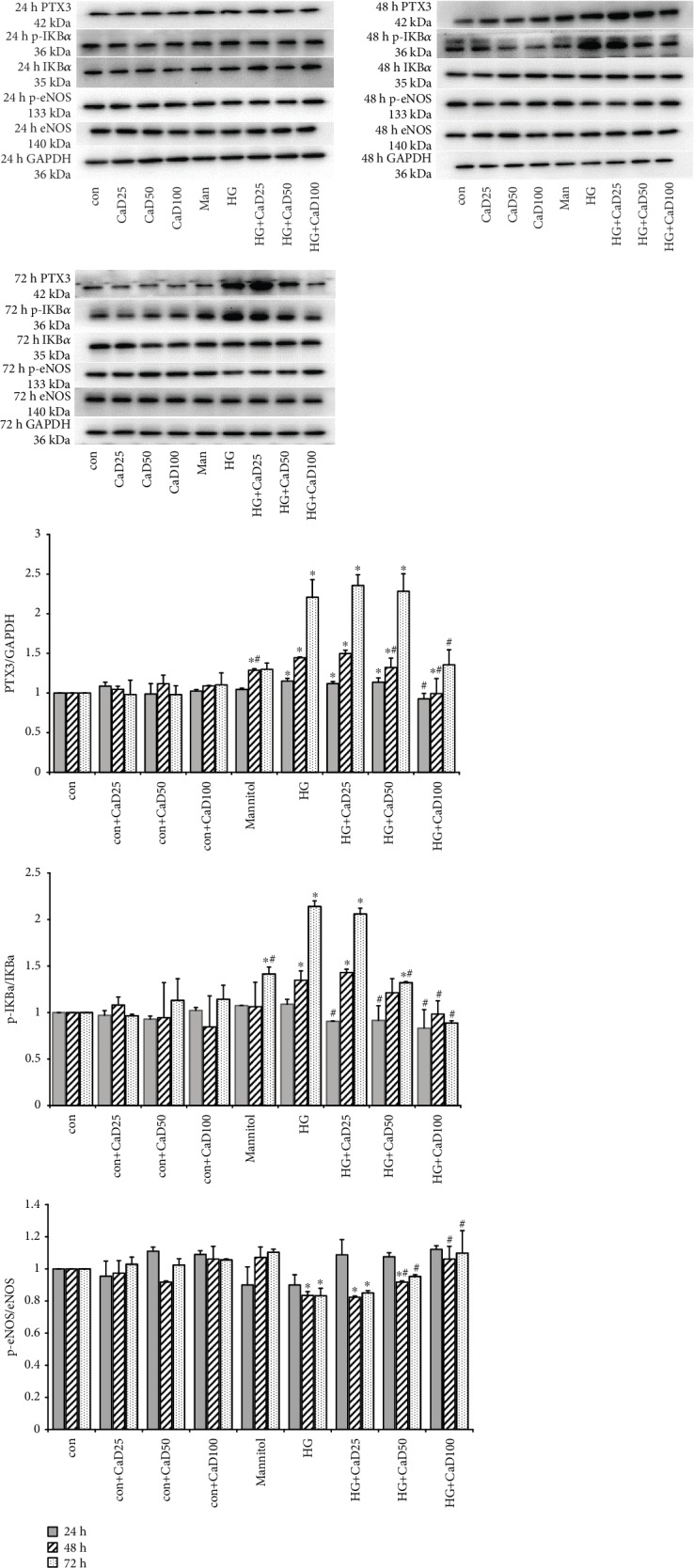
CaD inhibits the increased expression of PTX3 and p-IKBa/IKBa but enhances the decreased expression of p-eNOS/eNOS induced by high glucose; con: control; Man: mannitol; HG: high glucose; CaD25: calcium dobesilate 25 *μ*mol/L; CaD50: calcium dobesilate 50 *μ*mol/L; CaD100: calcium dobesilate100 *μ*mol/L; ^∗^compared with the control group, *P* < 0.05; ^#^compared with the HG group, *P* < 0.05).

The levels of PTX3, p-IKBa/IKBa, and p-eNOS/eNOS were detected in HUVECs. High glucose stimulation (35 mmol/L) increased the protein expression of PTX3 and p-IKBa/IKBa in a time-dependent manner (48 hours and 72 hours). The expression of p-eNOS/eNOS was reduced by high glucose in a time-dependent manner (48 hours and 72 hours). Different concentrations of CaD (25 *μ*mol/L, 50 *μ*mol/L, and 100 *μ*mol/L) were put into the control group and high glucose group for different periods of time (24 hours, 48 hours, and 72 hours). After CaD treatment, the abnormally increased expression of PTX3 and p-IKBa/IKBa was reduced, while the expression of p-eNOS/eNOS was increased. The control groups were cultured with 5.5 mmol/L glucose and different concentrations of CaD (Figure 2).

#### 3.1.3. TPCA-1 Blockade of the NF-*κ*B Signaling Pathway Decreases the High Glucose-Induced Increase in PTX3 Expression but Increases the High Glucose-Induced Decrease in p-eNOS/eNOS Expression in a Dose-Dependent Manner

The NF-*κ*B pathway is blocked by the IKK-2-specific inhibitor TPCA-1. TPCA-1 was tested at two concentrations (0.5 and 1.0 mmol/L) and was added to the culture dish 2 hours after the addition of high glucose medium (glucose concentration at 35 mmol/L). Control groups were established with the same inhibitor concentration (glucose concentration at 5.5 mmol/L+TPCA-1 at 0.5 and 1.0 mmol/L). The expression of PTX3 and p-eNOS/eNOS was measured after treatment for 48 hours.

**Figure 3:**
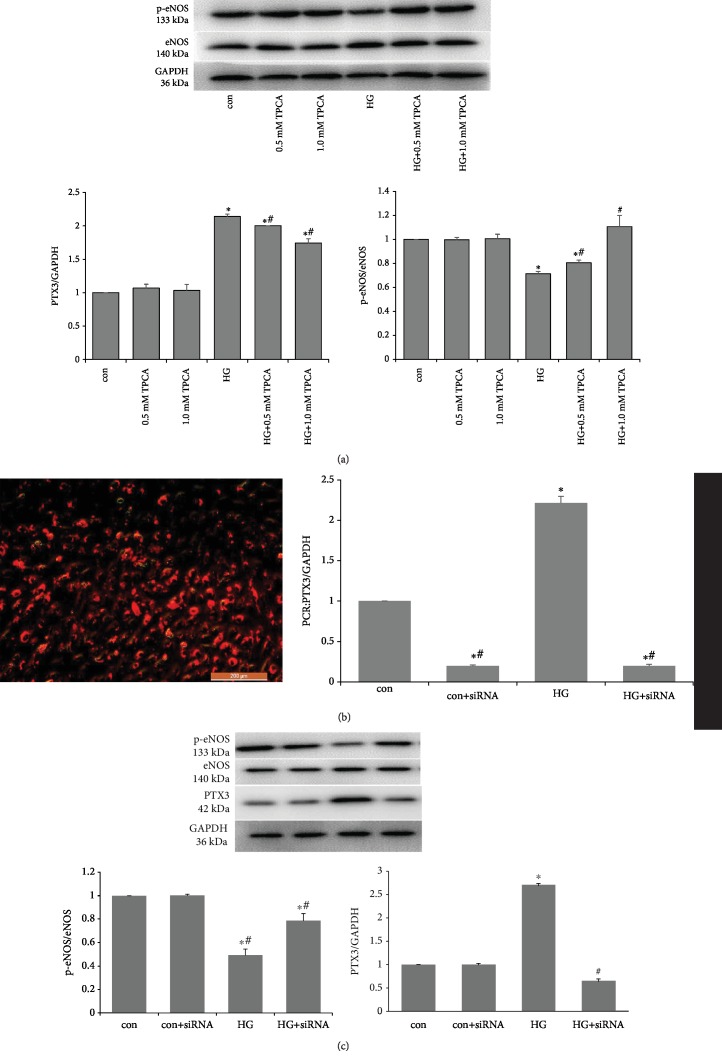
PTX3 is related to eNOS in vitro. (a) With the addition of TPCA-1, expression of PTX3 and p-eNOS/eNOS protein improved and was dose-dependent. (b) siRNA interference can effectively silence the expression of PTX3 in HUVECs (immunofluorescence showed Cy3 labeling control group and real-time PCR). (c) After PTX3 was silenced by siRNA, the expression of p-eNOS/eNOS protein was significantly higher than that in the HG group. con: control; HG: high glucose; ^∗^compared with the control group, *P* < 0.05; ^#^compared with the HG group, *P* < 0.05).

After blockade of the NF-*κ*B pathway by TPCA-1, the increased expression of PTX3 protein induced by high glucose was decreased in a dose-dependent manner, while the reduced expression of p-eNOS/eNOS induced by high glucose was increased in a dose-dependent manner. The expression of PTX3 and p-eNOS/eNOS in the control group did not change significantly (Figure 3(a)).

#### 3.1.4. siRNA Silencing of the PTX3 Gene Increases p-eNOS/eNOS Expression

After 24 hours of transfection with Cy3-labeled control siRNA, red fluorescence was observed in HUVECs under an inverted fluorescence microscope (Figure 3(b)). Real-time PCR analyses showed that PTX3 mRNA was significantly reduced in HUVECs 48 hours after transfection with PTX3 siRNA (Figure 3(b)). These results indicated that siRNA interference effectively silenced the expression of PTX3 in HUVECs.

After 48 hours of siRNA-mediated silencing of PTX3, the p-eNOS/eNOS protein expression of HUVECs in the high glucose environment was significantly increased compared to that in the cytoplasmic fraction of HUVECs in the high glucose control group. There was no significant change in the control group (Figure 3(c)).

### 3.2. In Vivo Studies

#### 3.2.1. CaD Reduces the Expression of PTX3 and p-IKBa/IKBa but Increases the Expression of p-eNOS/eNOS in the Kidneys of db/db Mice

Western blot results showed that the expression of PTX3 and p-IKBa/IKBa increased in the kidney tissues of db/db mice, while the expression of p-eNOS/eNOS was decreased.

**Figure 4:**
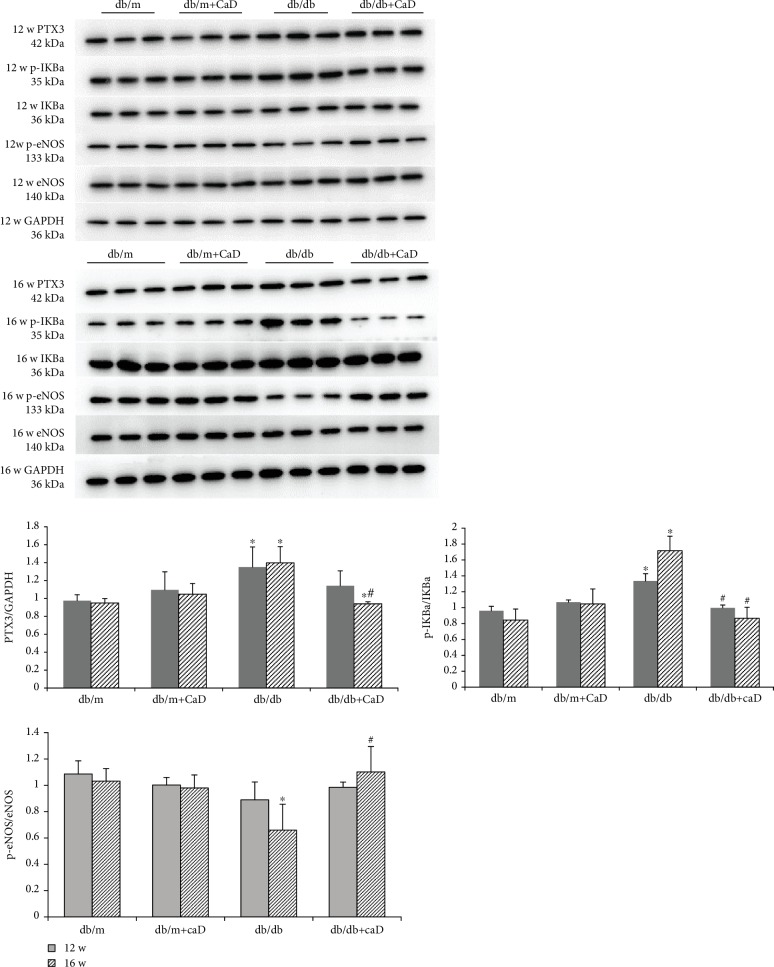
Calcium dobesilate reduced the expression of PTX3 and p-IKBa/IKBa protein in db/db mice but augmented that of p-eNOS/eNOS. ^∗^Compared with the db/m group, *P* < 0.05; ^#^compared with the db/db group, *P* < 0.05.

The expression of PTX3 and p-IKBa/IKBa in db/db mice treated with CaD was significantly lower than that in untreated db/db mice, while the expression of p-eNOS/eNOS was increased (Figure 4).

#### 3.2.2. Injection of Adenovirus-Encapsulated siRNA into db/db Mice Effectively Silences PTX3

**Figure 5:**
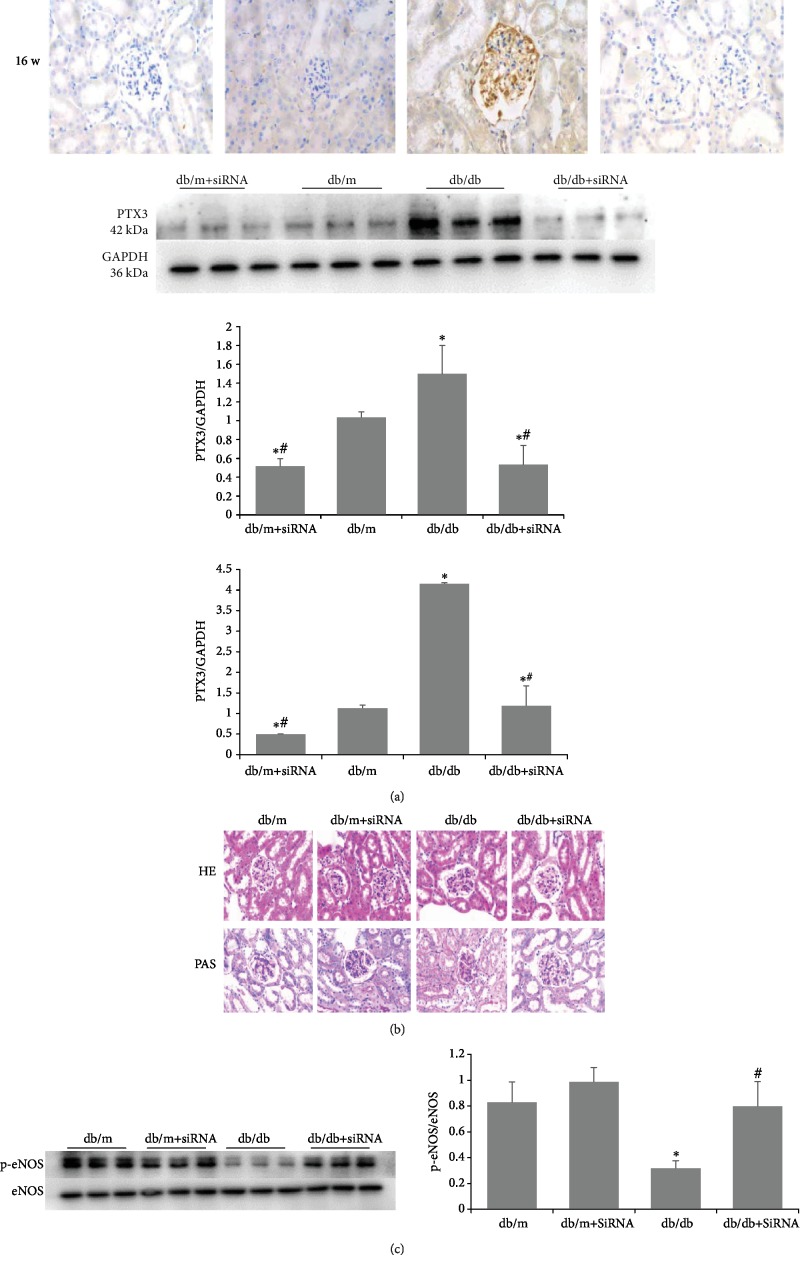
PTX3 is related to eNOS in vivo. (a) PTX3 was silenced by siRNA: PTX3 immunofluorescence staining results; Western blot and real-time PCR results. (b) Pathological changes of kidney (HE staining ×400) (PAS staining ×400). (c) The expression of p-eNOS/eNOS increased in db/db mice after silencing PTX3 gene. ^∗^Compared with the db/m group, *P* < 0.05; ^#^compared with the db/db group, *P* < 0.05.

Starting at the 8th week, db/db mice and db/m mice were injected with adenovirus via the tail vein at a dose of 2 × 10^8^ PFU per injection once every two weeks. The control group was injected with the same volume of normal saline. The mice were sacrificed after the 16th week. Immunohistochemical staining of PTX3 revealed that db/db mice injected with siRNA showed a significant decrease in PTX3 expression in the glomeruli. Real-time PCR and Western blot results were consistent with the immunohistochemical results. These findings indicated that injection of db/db mice with adenovirus-encapsulated siRNA effectively silenced the PTX3 gene (Figure 5(a)).

#### 3.2.3. PTX3 Silencing Improves Urinary Protein and Renal Function in db/db Mice

In PTX3-silenced db/db mice, 24-hour urinary albumin was significantly reduced at 16 weeks of age compared to that of db/db mice of the same age (db/db+siRNA: 200.08 ± 14.07 *μ*g/24 h; db/db: 400.08 ± 35.49 *μ*g/24 h; *P* < 0.05).

In terms of renal function, PTX3-silenced db/db mice showed a significant decrease in cystatin C at 16 weeks of age compared to db/db mice of the same age (db/db+siRNA: 652.99 ± 55.20 *μ*g/L; db/db: 922.33 ± 48.16 *μ*g/L; *P* < 0.05). However, there was no significant difference in serum cystatin C between the siRNA and control db/m groups.

siRNA injection had no effect on body weight, blood glucose, serum cholesterol, or triglycerides in db/db mice (Table 2).

#### 3.2.4. PTX3 Silencing Reduces the Glomerular Extracellular Matrix in db/db Mice

At 16 weeks of age, glomerular hypertrophy, mesangial matrix expansion, and increased glomerular extracellular matrix were observed in db/db mice, as shown by HE staining. After the PTX3 gene was silenced, the above changes were alleviated.

The periodic acid-Schiff (PAS) staining results were consistent with the hematoxylin-eosin (HE) staining results. Under PAS staining, the glomerular mesangial matrix was purplish red, and the rest of the cells presented no staining, except for deep blue staining of the nucleus. At 16 weeks of age, PAS staining in db/db mice showed mesangial matrix expansion and a significant increase in glomerular extracellular matrix. The above changes were alleviated after silencing the PTX3 gene (Figure 5(b)).

#### 3.2.5. PTX3 Silencing Increases the Expression of p-eNOS/eNOS in db/db Mice

After siRNA silencing of PTX3, the expression of p-eNOS/eNOS in the kidneys of db/db mice was increased. There was no significant change in the control group (Figure 5(c)).

## 4. Discussion

Our study showed that high glucose might promote the expression of PTX3 by activating the IKK/IKB/NF-*κ*B pathway, which interfered with the phosphorylation of eNOS at Serine 1177, thereby reducing the production of nitric oxide and leading to endothelial cell dysfunction. CaD protection might be partially mediated through the NF-*κ*B/PTX3/eNOS pathway.

A meta-analysis of diabetic retinopathy showed that CaD significantly improves fundus microaneurysms and fundus hemorrhage, reduces whole blood viscosity and plasma viscosity, decreases blood lipid levels, reduces intraocular pressure, and effectively treats diabetic retinopathy at the systemic and local levels. Thus, CaD is widely used to treat this disease [18]. Meanwhile, our past study also found that CaD could suppress high glucose-induced PTX3 overexpression in cultured HUVECs. This finding indicated that CaD protected endothelial cells partly by ameliorating high glucose-induced inflammation [21]. In diabetic retinopathy, studies have confirmed that CaD may protect the retina and reduce chemotaxis and aggregation of retinal inflammatory cells by inhibiting NF-*κ*B and p38 MAPK activity [19]. Among the many microvascular complications of diabetes, diabetic nephropathy and diabetic retinopathy usually coexist, develop in parallel, and have a similar pathogenesis [20]. Although studies have suggested that CaD may reduce microalbuminuria in patients with DKD by reducing plasminogen activator inhibitors, the underlying mechanism is not clear [22]. The present study confirmed that CaD inhibits high glucose-induced activation of the NF-*κ*B pathway in HUVECs, which was consistent with past retinal research results. In db/db mice, the kidney expression of p-IKBa/IKBa in the CaD group was decreased. After treatment with CaD, the expression of p-IKB*α*/IKB*α* and PTX3 was decreased, but the expression of p-eNOS/eNOS was increased in the HUVECs and kidney tissues of db/db mice stimulated by high glucose. After CaD treatment, the urinary protein and renal function of db/db mice were improved. These results suggested that CaD may inhibit the expression of PTX3 by regulating the IKK/IKB/NF-*κ*B pathway, thereby improving endothelial cell function and protecting renal function. These findings also indicated that CaD may improve kidney endothelial dysfunction in diabetic nephropathy by inhibiting the inflammatory reaction, thus protecting the kidney.

Microinflammation and chronic activation of innate immunity are important causes of diabetic nephropathy [23, 24]. Several studies have suggested that many inflammatory factors, such as tumor necrosis factor alpha (TNF-*α*), interleukin- (IL-) 1, IL-6, and IL-18, are highly expressed in the kidneys of diabetic animal models [25, 26]. Serum levels of IL-18 and TNF-*α* are higher in diabetic patients than in control subjects and were positively correlated with albuminuria [27, 28]. PTX3 is synthesized in extrahepatic tissues and cells, including atherosclerotic plaques, adipose tissue, vascular endothelial cells, and macrophages. Studies have shown that renal peritubular endothelial cells express PTX3 in a mouse model [29]. In humans, renal proximal tubule endothelial cells, mesangial cells, and kidney fibroblasts all secrete PTX3 [30, 31]. In addition, elevated levels of PTX3 were found in the plasma of patients with chronic kidney disease and diabetic nephropathy [32, 33]. PTX3 may be associated with kidney damage, but the pathogenesis of PTX3 in diabetic nephropathy is still unclear. In fact, PTX3 is also associated with endothelial cell dysfunction, and inhibition of PTX3 expression may improve endothelial cell function [34, 35]. Therefore, PTX3 is closely related to endothelial cell function and diabetic nephropathy, and PTX3 may play an important role in renal damage caused by endothelial cell dysfunction. The state of inflammation may also be the cause of endothelial disorders. Our study demonstrated that high glucose induces the upregulation of inflammatory factors in vivo and in vitro. High glucose upregulated PTX3 expression in a time- and concentration-dependent manner in vitro. Plasma PTX3 levels were also significantly elevated in db/db mice.

Nitric oxide is a major determinant of endothelial cell relaxation [16]. High glucose leads to a decrease in the p-eNOS/eNOS ratio and a decrease in NO synthesis, which triggers overexpression of VEGF-A and results in abnormal proliferation of endothelial cells [36]. Our study found that high glucose caused a decrease in the p-eNOS/eNOS level in endothelial cells in a time-dependent and glucose concentration-dependent manner. In vivo results suggested that the p-eNOS/eNOS levels in 16-week-old db/db mice were significantly reduced compared to the levels in control mice. CaD improved the high glucose-induced decrease in p-eNOS/eNOS levels in a concentration-dependent manner. These findings suggested that under high glucose conditions, CaD may improve the activity of eNOS.

PTX3 is closely related to eNOS, and PTX3 damages the nitric oxide synthase/nitric oxide pathway through various pathways [17] [37–39]. The activation of eNOS first requires phosphorylation at the Tryptophan 1177 site and then dissociation from caveolin-1 (cav-1) to form eNOS dimers, which mediate nitric oxide production. Studies have shown that PTX3 inhibits the NOS/NO pathway by inhibiting eNOS phosphorylation, which causes detachment from cav-1 [37]. In vascular endothelial cells, eNOS binds primarily to cav-1, which is located in the specific structure of caveolae on the cell membrane [38]. cav-1 binds to eNOS, which maintains the eNOS monomer structure and negatively regulates the enzymatic activity of eNOS [39]. After stimulation of blood vessels with PTX3, coimmunoprecipitation experiments demonstrated that the cell membrane structure was destroyed and that cav-1 was transferred to the cytoplasmic region of the cells where it is continuously bound to eNOS, which affected formation of the eNOS active form—eNOS dimer [38]. Carrizzo et al. also found that PTX3 induces vascular endothelial dysfunction through a P-selectin/matrix metalloproteinase-1(MMP-1) pathway. PTX3 significantly blunted nitric oxide production through the MMP-1 pathway [17]. In the present study, high glucose increased PTX3 expression and decreased p-eNOS/eNOS expression. The expression of p-eNOS/eNOS in the HUVECs and renal tissues of db/db mice was increased after siRNA silencing of the PTX3 gene. CaD treatment decreased PTX3 expression and increased p-eNOS/eNOS expression both in vivo and in vitro. These findings demonstrated that high glucose may interfere with the phosphorylation of eNOS at Serine 1177 by promoting the expression of PTX3, which reduces the production of nitric oxide, thereby leading to endothelial damage induced by endothelial cell dysfunction. CaD may protect endothelial function through this pathway.

The present study suggested that CaD has therapeutic potential by improving microinflammation and endothelial function in patients with diabetic nephropathy. PTX3 may also be a potential therapeutic target for the treatment of diabetic nephropathy.

## 5. Conclusions

CaD may inhibit the expression of PTX3 by altering the IKK/IKB/NF-*κ*B pathway, thereby improving endothelial dysfunction on the cellular level. PTX3 may be a potential therapeutic target for DKD.

## Figures and Tables

**Table 1:** The sequences of primers and siRNAs used in this study.

Gene	Sequence
PTX3 (human)	Forward: 5′CATCTCCTTGCGATTCTGTTT3′ Reverse: 5′CCATTGTCTATTTCGTTGTCCA3′

GAPDH (human)	Forward: 5′AGAAGGCTGGGGCTCATTTG3′ Reverse: 5′AGGGGCCATCCACAGTCTTC3′

PTX3 (mouse)	Forward: 5′TCTGTTCCTGAGGGTGGACT3′ Reverse: 5′CCGATCCCAGATATTGAAGC3′

GAPDH (mouse)	Forward: 5′GGTGAAGGTCGGTGTGAACG3′ Reverse: 5′CTCGCTCCTGGAAGATGGTG3′

siRNA-PTX3 (human)	5′GGAAGCGTGCATCCAGTGAGAC3′

siRNA-PTX3 (mouse)	5′GCCACAGATGTATTAAACA3′

**Table 2:** Comparison of basic data between injected siRNA mice and control group.

	16-week-age			
	db/m	db/m+siRNA	db/db	db/db+siRNA
Weight (g)	26.06 ± 2.29	25.65 ± 2.23	56.25 ± 1.87	54.18 ± 2.48
Blood sugar (mmol/L)	3.63 ± 0.49	3.56 ± 0.65	13.75 ± 5.87	13.64 ± 4.92
Cholesterol (nmol/L)	16.94 ± 1.36	17.95 ± 0.98	19.84 ± 0.73	17.12 ± 1.83
Triglyceride (nmol/L)	27.85 ± 0.60	26.97 ± 2.85	34.91 ± 1.88	32.74 ± 1.68
24-hour urinary albumin (*μ*g/24 h)	19.49 ± 10.90	19.93 ± 8.97	400.08 ± 35.49^∗^	200.08 ± 14.07^∗#^
Cystatin C (*μ*g/L)	695.27 ± 52.48	698.26 ± 54.48	922.33 ± 48.16^∗^	652.99 ± 55.20^#^

^∗^Compared with the db/m group, *P* < 0.05; ^#^compared with the db/db group, *P* < 0.05.

## Data Availability

The data used to support the findings of this study have not been made available because they belong to certain parts of the National Natural Science Foundations of China. There are other relevant important data that have not been published yet.
